# Congenital central hypoventilation syndrome mimicking mitochondrial disease

**DOI:** 10.1002/ccr3.1320

**Published:** 2018-01-19

**Authors:** Kitiwan Rojnueangnit, Maria Descartes

**Affiliations:** ^1^ Department of Pediatrics Faculty of Medicine Thammasat University Pathumthani Thailand; ^2^ Department of Genetics University of Alabama at Birmingham Birmingham Alabama; ^3^ Department of Pediatrics University of Alabama at Birmingham Birmingham Alabama

**Keywords:** Congenital central hypoventilation syndrome, episodic of respiratory failure, milder later‐onset, *PHOX2B*

## Abstract

Later‐onset congenital central hypoventilation syndrome (LO‐CCHS) does not present only breathing problems but can be present as episodic multiple organs involvement. Our unique case demonstrated LO‐CCHS should be considered in the differential diagnosis of mitochondrial diseases and having nontypical polysomnography result.

## Introduction

Congenital central hypoventilation syndrome (CCHS: MIM 209880) is a rare unique respiratory center defect of absent or decreased response to hypoxemia and/or hypercapnia, leading to alveolar hypoventilation. Previously, the incidence was thought to be very low. It appears that the incidence of CCHS is no longer as rare as anticipated but is likely underestimated due to lack of recognition, clinical variation, and decreased penetrance. There is no population study on incidence. Very few studies 1, 2 have tried to estimate incidence by identifying the positive mutation in symptomatic cases, which might not include all CCHS cases. At this point, more than 1000 individuals confirmed the diagnosis worldwide 3, 4. The typical form usually presents in newborns with hypoventilation, and the later‐onset form presents in infancy or later with milder presentation, during sleep, or after triggers such as postoperation under general anesthesia or postrespiratory tract infection.

*PHOX2B* (paired‐like homeobox 2B), located at 4p12, is only the disease‐causing gene for CCHS 5, 6, encoding the homeobox domain transcription factor of 314 amino acids. PHOX2B, a transcription factor, is not expressed during autonomic nervous system development and pivotal in the development of most relays of the autonomic nervous system, including all autonomic neural crest derivatives 7, but Phox2b remains widely expressed in the adult rat brain. Its expression persists in most neuronal groups whose embryogenesis depends on Phox2b expression 8. More than 90% of the mutation occurs in polyalanine repeat (PAR) region 1, 2, 6, 9, which normally has 20 PARs. CCHS has been reported in heterozygous mutations of 24–33 PARs 1, 6, 10, 11, 12. The rest of the mutation does not occur in PAR region but have been described includes frameshift, nonsense, missense, in‐frame, stop codon alterations, and large deletions 3, 9. There is a correlation between PAR length and the severity of the respiratory phenotype 10, 13, a later onset usually found in the heterozygous of 24 PARs genotype, and some of 25 PARs; however, the 25 PARs patient could present as either a typical form or a later‐onset form, of which the genotype–phenotype correlation is still unclear 2, 10, 14. The expression studies demonstrated the PARs expansion leads to mis‐localize of the mutant PHOX2B protein and disrupted the normal protein function by aggregation with the mutant 15, 16. The most individuals with CCHS are heterozygous for de novo pathogenic variant. Nevertheless, up to 10% may have asymptomatic parents with mosaic mutation 6, 16. The recurrence risk for an affected individual is 50%.

Most patients with LO‐CCHS need mechanical ventilation only during sleep, whereas a subset of severely affected individuals require continuous mechanical ventilation 3, 17.

Mitochondrial disease is usually in the differential diagnosis of multiple organ involvements with/without episodic manifestation. It is difficult to rule out given the sheer variety of clinical manifestations and investigations. As a consequence, there are patients who receive treatment and are monitored as possible mitochondrial disease cases but have never been confirmed. Here, we report an 18‐month‐old girl with CCHS who presented as mitochondrial disease but revealed all negative test results.

## Clinical Report

The patient, an 18‐month‐old African American girl, was transferred to our institution (pediatric intensive care unit) for history of weakness, loss of motor function, transaminitis, pleural effusion, and respiratory failure. She was the second child born to a 43‐year‐old G5P1 mother by repeat cesarean section at term. The mother had history of three‐first‐trimester miscarriages and one stillborn and a 51‐year‐old father. No complications were reported during the pregnancy and delivery with a birthweight of 2.9 kg (25th centile) and a length of 53 cm (75th centile). She was previously healthy with no known underlying disease; her development was normal for her age. Past medical history pertinent for patient is a carrier for sickle cell anemia.

Three weeks prior to admission (1 week after routine immunization at the age of 18 months), she had an upper respiratory tract infection without fever. One week prior to admission, she was noted with abdominal distension, decreased appetite, refusal to walk, and lethargy. Her symptoms progressed to acute cardio‐respiratory failure requiring intubation with positive pressure ventilation support. Physical examination demonstrated afebrile, generalized muscle weakness with intact consciousness, cardiomegaly, hepatomegaly, transaminitis, pleural effusion, and ascites. Her venous blood gas revealed hypercapnia (P_v_CO2 79 mmHg), and her oxygenation was low in 70–80% (room air). Chronic obstructive hypoventilation from hypotonia and muscle weekness was suspected. However, her clinical symptoms improved, and extubation was performed on day 5. She recovered completely within 2 weeks. Viral infection was suspected without any confirmation tests, including hepatitis A, parvovirus, adenovirus, enterovirus, and cytomegalovirus. Initial metabolic workup included plasma amino acids, urine organic acids, and plasma acylcarnitine profile; they were all unremarkable.

Three months later, at the age of 21 months, she developed a second episode of acute cardio‐respiratory failure. She had 2 days history of decreased appetite and a one‐day history of periorbital swelling. On admission, cardiac failure with pulmonary hypertension was found. She was also noted to have generalized muscle weakness. The echocardiogram showed the right ventricular hypertrophy and right atrium enlargement. Lung biopsy was performed, revealing mild pulmonary arterial hypertensive changes. Brain MRI showed multifocal small signal dropout, suggesting a collection of iron. Intubation was required for a week, and after extubation, she had desaturation when asleep; therefore, a polysomnography was performed. The study showed hypoxia, hypoventilation with hypercapnia (P_v_CO2 range from 44 to 62 mmHg), and hypoventilation was documented. Her clinical outcome improved, and she was discharged from the hospital 3 weeks later with noninvasive respiratory support during sleep at home.

**Figure 1:**
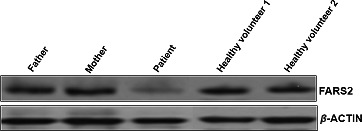
FARS2 protein expression in leukocytes using Western blotting, upper row showing a half of FARS2 protein expression in the patient when compared with her parents and two healthy controls; lower row showing an *β*‐actin expression used as an internal control.

Mitochondrial disease was suspected after patient's second episode given multisystemic involvement. Her evaluation was extensive. Muscle biopsy revealed normal histology, but mitochondrial oxidative phosphorylation studies unveiled low oxidative phosphorylation in complex I (3%). Chromosome microarray revealed a microdeletion 270 kb of the short arm of chromosome 6 (6p25.1), involved in *FARS2*, a nuclear mitochondrial gene. As a mitochondrial disease was suspected, the patient was followed and empirically treated as having a mitochondrial disease and started on several mitochondrial cofactors (carnitine, coenzyme Q, thiamine, leucovorin, riboflavin, vitamin C, pantothenic acid, alpha‐lipoic acid, and creatine). Molecular analysis/sequencing of the remaining allele for *FARS2* was negative, and functional enzyme analysis confirmed happloinsufficiency of FARS2 protein as anticipated, when compared with her parents and healthy controls (Fig. 1). All 37 mitochondrial and 200 mitochondrial nuclear genes including the nuclear genes for oxidative phosphorylation complex I were sequenced investigated, and no known deleterious mutations were detected. During her clinical course, she improved. Her right ventricular hypertrophy and atrium enlargement resolved.

*PHOX2B* was tested for CCHS due to several instances of hypercapnia (with maximum PCO_2_ 80 mmHg) on polysomnography, although it did not demonstrate definitive central sleep apnea because of hypoxia (with baseline room air SpO2 87–92%) which was found during sleep study. The results revealed c.741_755dup (p. Ala256_Ala260dup), leading to a heterozygous expansion of the polyalanine to 25 repeats (genotype 20/25). Parental testing was recommended but not carried out, therefore, de novo mutation could not be confirmed. Her sister was proved to be negative for the polyalanine expansion.

She was initially treated with bilevel positive airway pressure (Bi‐PAP) mode only while sleeping that was later converted to continuous positive airway pressure (C‐PAP) and did well with resolution of her right heart issues. Mitochondrial cofactors were discontinued, and she was up to date on recommended vaccines.

## Discussion

We report a previously healthy girl with two episodes of acute respiratory failure with multiorgan involvement suggestive of mitochondrial disorder. In Addition, the microdeletion of the short arm on chromosome 6 (6p25.1) involving *FARS2*, and decreased oxidative phosphorylation of the complex I, led to suspicion of mitochondrial disease as the most likely diagnosis. However, after the full and in‐depth investigation for mitochondrial disease, all tests remained uninformative. Finally, *PHOX2B* revealed the heterozygous mutation for PAR, confirming the diagnosis of LO‐CCHS. The challenge in this case was the nontypical polysomnography of central hypoventilation pattern; therefore, CCHS should be still in the differential diagnosis for hypoventilation patient.

The phenotype of our patient was similar to other patients with LO‐CCHS, which was previously reported in heterozygous 24 PAR 10, 11 and some of 25 PAR 2, 18, 19, 20, 21. Her ascites, pleural effusion, hepatomegaly, as well as pulmonary hypertension, right ventricular hypertrophy, and right atrium enlargement could be explained from pulmonary and cardiac failure. Generalized muscle weakness and hypotonic condition could be the transient effect from brain encephalopathy.

Neurocognitive impairment has been reported in patients with CCHS; however, in the patients with 20 of 25 genotypes; most are not be affected 2, 17, 22 if they received proper management and adequate brain oxygen. Most of the later‐onset patients presented after sedation or general anesthesia, or after severe respiratory infection/illness with inadequate respiratory effort as a clinical phenotype 11, 18. However, in some cases, there has not been a definitive or precise precipitating factors 18, as well as in this case.

According to treatment for CCHS, this case showed the reversible process of pulmonary hypertension and right‐sided heart enlargement and hypertrophy remodeling after adequate and proper treatment.

## Conclusion

In patients who present with multiple organs involvement and are suspected to have mitochondrial diseases, LO‐CCHS should be one of the differential diagnosis. *PHOX2B* molecular testing is mandatory to confirm the diagnosis of CCHS. Early diagnosis and treatment is one method of protection against neurocognitive impairment and sequelae of cardiac and pulmonary complications. Therefore, awareness is the major key for the best outcome.

## Authorship

KR: followed up with the patient and performed genetic counseling, coordinated with Western blot testing, wrote the manuscript, and reviewed the literature. MD: supervised, sending genetic tests and follow up with the patient, revised the manuscript, and reviewed the literature.

## Conflict of Interest

The authors declare no conflict of interest.
